# Using serum placenta growth factor could improve the sensitivity of colorectal cancer screening in fecal occult blood negative population: A multicenter with independent cohort validation study

**DOI:** 10.1002/cam4.2216

**Published:** 2019-05-07

**Authors:** Shu‐Chen Wei, Po‐Nien Tsao, Yu‐Ting Wang, Been‐Ren Lin, Deng‐Chyang Wu, Wen‐Sy Tsai, Jinn‐Shiun Chen, Jau‐Min Wong

**Affiliations:** ^1^ Department of Internal Medicine National Taiwan University Hospital and College of Medicine Taipei Taiwan; ^2^ Department of Pediatrics National Taiwan University Children’s Hospital and National Taiwan University Taipei Taiwan; ^3^ The Research Center for Developmental Biology and Regenerative Medicine National Taiwan University Taipei Taiwan; ^4^ Department of Medical Research National Taiwan University Hospital Taipei Taiwan; ^5^ Department of Surgery National Taiwan University Hospital and College of Medicine Taipei Taiwan; ^6^ Division of Gastroenterology, Department of Internal Medicine Kaohsiung Medical University Hospital Kaohsiung Taiwan; ^7^ Division of Colon and Rectal Surgery Chang Gung Memorial Hospital Linkou Taiwan; ^8^ School of Medicine Chang Gung University Taoyuan Taiwan

**Keywords:** colorectal cancer, FOBT, serum PlGF

## Abstract

**Background:**

Colorectal cancer (CRC) is one of the most common cancers worldwide. Screening for CRC using the fecal occult blood test (FOBT) is feasible and useful for decreasing disease‐related mortality; however, its sensitivity and compliance are unsatisfactory.

**Methods:**

This study examined the efficacy of using serum placenta growth factor (PlGF) for a novel CRC screening strategy. To investigate a potential novel screening tool for CRC, we compared the sensitivity, specificity, positive predictive value, and negative predictive value of the FOBT, serum PlGF, and their combination through an examination of two independent cohorts and validation using the second cohort. All the patients and control group received the colonoscopy and FOBT, the colonoscopy was used as the gold standard for the result.

**Results:**

Serum PlGF levels were significantly increased in CRC patients (16.8 ± 11.4 pg/mL) compared with controls (12.0 ± 11.2 pg/mL). The predictive model that used the serum PlGF level alone was as effective as the FOBT (AUC: 0.60 vs 0.68, *P* = 0.891), and it had significantly higher sensitivity than the FOBT (0.81 vs 0.39). In addition, we found serum PlGF level has a good value for predicting CRC patients in those FOBT negative populations. Finally, combining serum PlGF level and the FOBT improved the predictive power and demonstrated satisfactory sensitivity (0.71) and specificity (0.71). This result was confirmed and validated in the second independent cohort. Furthermore, no matter the stages (early/advanced) and the location (distal/proximal) of CRC, the efficacy of serum PlGF and the combined model remained quite stable.

**Conclusion:**

Serum PlGF level is a potential alternative screening tool for CRC, especially for those who are reluctant to stool‐based screening methods and who were tested as negative FOBT. In addition, combining serum PlGF level and the FOBT could increase the power of CRC screening.

## 1 INTRODUCTION

Colorectal cancer (CRC) is the third most common cancer globally, with over 1.3 million cases that account for 10% of all cancers; it results in roughly 700 000 deaths per year.^1^ According to the pathogenesis, most incidences of CRC develop from adenomas over many years; thus, screening can be highly beneficial and early detection is much more likely than for most other types of cancers. Randomized trials have demonstrated the positive effect of screening with stool tests and endoscopic examinations on CRC incidence and mortality rates.^2^, ^3^, ^4^, ^5^, ^6^, ^7^ Therefore, screening programs are currently being implemented in an increasing number of countries.^8^

The tools currently available for CRC screening include the fecal occult blood test (FOBT), flexible sigmoidoscopy, and colonoscopy,^6^ each of which has its distinct advantages and disadvantages. Meta‐analyses have indicated that the guaiac FOBT decreased CRC‐related mortality by 16%, and sigmoidoscopy decreased CRC‐related mortality by 30%.^6^, ^9^ Yet, a gap remains between screening efficacy and expectations, and a bottleneck status seems to persist with respect to the CRC screening using these modalities. Evidence indicates that the patients exhibiting low compliance with a recommended CRC screening program are at a high risk for CRC.^10^

The FOBT is a noninvasive and relatively low‐cost test, but its compliance rate is as low as 40%‐50%.^11^ Other disadvantage of using the FOBT for CRC screening is its low sensitivity (especially for the proximal colon lesion), which limited the effect of CRC screening.^12^ In addition, although the strategy of using repetitive FOBTs have been a popular strategy, but the problem of compliance still remains, and its cost is also high.^13^ Therefore, we need to find another approach to improve the CRC screening value, especially for the FOBT negative and also those reluctant to stool‐based screening populations.

Colonoscopy is currently the gold standard for CRC screening; it exhibits high sensitivity and specificity (sensitivity and specificity greater than 98%).^10^ However, the implementation of this invasive procedure for population‐wide screening is limited because of several disadvantages, such as high cost, limited resources, and low compliance.^14^, ^15^, ^16^ Because it is an invasive examination procedure, even with careful technique, it still entails a 1/1000 to 1/10 000 risk of perforation injury. Furthermore, many patients are reluctant to undertake the necessary preexamination colon preparations, resulting in low compliance.^10^

The effectiveness of screening depends on the appropriateness and acceptance of the modality. The ideal modality for screening should have high acceptance and compliance, high sensitivity and high specificity, and low invasiveness and should be cost effective. Thus, expending effort to develop a simple and non‐ or minimally invasive screening model is a laudable goal. Studies have investigated the use of combined novel biomarkers, such as epigenetic biomarkers,^17^ proteomic markers and fecal DNA markers,^18^, ^19^ and metabolites,^20^ for screening. Because regular blood tests are widely accepted, discovery of an appropriate serum marker could form the base for a novel screening tool and increase compliance. A blood‐based test that could be easily integrated into routine examinations and requires minimal preparation might therefore be a highly attractive alternative to minimally invasive CRC screening; however, we first must discover the appropriate biomarker.

Placenta growth factor (PlGF) is a member of the vascular endothelial growth factor (VEGF) family that binds to VEGF receptor (VEGFR)‐1 but not VEGFR‐2; it may be structurally and functionally associated with VEGF,^21^, ^22^ PlGF has been discovered to be associated with disease progression and survival in several human tumor diseases.^23^, ^24^, ^25^, ^26^, ^27^ Our previous studies have also demonstrated that the PlGF is significantly high in CRC tissues and that it is associated with tumor progression and disease prognosis.^28^, ^29^, ^30^ This finding has been reproduced in other studies.^31^, ^32^, ^33^ In addition, preoperative blood PlGF level has been demonstrated to be high in CRC patients.^28^, ^32^ This suggests that the level of serum PlGF may be a useful biomarker for CRC screening. However, its predictive power and sensitivity for CRC remains unclear.

It is also of major interest to determine the effectiveness of combining the blood‐based tests and the FOBT for detecting early‐stage CRC or its proximal location. Several blood‐based protein biomarkers, such as CEA, CA19‐9, and CA242, have been associated with the diagnosis of CRC; however, none of these proteins have individually been able to detect the majority of early‐stage or proximal CRC.^7^, ^34^ This study was aimed to evaluate the effectiveness of CRC screening using serum PlGF level alone and also focusing on its addictive effect for stool OB negative group, with a multi‐centers, two stages study.

## 2 MATERIALS AND METHODS

This study was approved by the Ethical Committee of the National Taiwan University Hospital, Chang Guan Memorial Hospital, and Kaohsiung Medical University Hospital. Two independent cohorts were analyzed in this study. Both cohort 1 and cohort 2 enrolled the patients (newly diagnosed CRC patients) and control group (healthy checkup who had the stool occult blood test and colonoscopy) consecutively during the study period. Both cohorts excluded those with previous cancer history, who had NSAIDs within 3 months.

Aligned with our daily clinical practice, patients in the first cohort were enrolled for a retrospective study; this cohort included clinically healthy controls who underwent guaiac FOBTs and colonoscopy as part of a general checkup. After obtaining informed consent, their clinical data and serum were collected. For the CRC patients group, all serum was collected after obtaining informed consent but before surgery or chemotherapy. Samples were collected from 2014 to 2015. Clinical staging of cancers was determined per the International Union against Cancer TNM classification. No presurgical chemotherapy or radiotherapy was administered to the colon cancer group, whereas patients with Stage III and Stage IV colon cancer were subjected to postoperative chemotherapy with 5‐fluorouracil and leucovorin.

Since smoking and family history were not available in the first retrospective cohort, therefore, a prospective cohort was conducted in order to get more parameters to adjusting the possible confounding factors and also to validate the first stage results. For the second cohort, a prospective study was conducted in the aforementioned three medical centers from January 2016 to December 2017. Patients in the CRC patients group were enrolled before receiving surgery or chemotherapy, and we also included family history and smoking status, which were lacking in the first cohort which was a retrospective study. A healthy control group was enrolled from the general checkup group.

### 2.1 Measurement of Serum PlGF

Five milliliters of blood from each individual was collected and all serum samples were stored at −80°C until use. The level of PlGF in serum was assayed using a standardized sandwich enzyme‐linked immunosorbent assay (ELISA; R&D Systems, Minneapolis, MN) in triplicate according to the protocol recommended by the manufacturer and our previous publication, the concentrations of PlGF were expressed as picograms per milliliter.^28^

### 2.2 Statistical analysis

The characteristics of the study population were represented as the mean ± standard deviation (SD) for continuous variables and frequency (percentage) for categorical variables. The differences between patients with CRC and the healthy controls were assessed using *t* tests and chi‐squared tests. The sensitivity and specificity of the prediction models were measured using logistic regressions.

Patients in the second cohort were randomly separated into a training set (N = 260) and a validation set (N = 260), and set up three predictive models according to FOBT, serum PlGF levels, and combined FOBT and PlGF levels. Youden's index of maximum potential effectiveness was used for the optimal cut‐point of each predictive model. Finally, we used these cut‐points of each predictive model to validate the sensitivity and specificity in the validation set.

All analyses were performed using SAS 9.4 (SAS Institute, Cary, NC), and *P* < 0.05 was considered statistically significant.

## 3 RESULTS

### 3.1 The FOBT test yielded a high false negative rate of CRC

In total, we collected 445 patients with CRC and 458 healthy controls in the first cohort. The CRC stage breakdown of these patients was as follows: Stage 0, 1 (0.2%); Stage I, 102 (23.0%); Stage II, 114 (25.7%); Stage III, 166 (37.4%); Stage IV, 61 (13.7%) (Table 1). The mean age at CRC diagnosis was 63.7 ± 12.7 years (Table 1), and the patients were predominantly male (59.33%). The mean age of the healthy control group was 55.3 ± 14.7 years, which was younger than the CRC group but consistent with real world experience (CRC screening begins at 50 years, and the mean age of CRC diagnosis is approximately 65 years). No difference was evident in the sex ratio or body mass index (BMI) between the healthy control group and patients with CRC. The positive rate for the FOBT was significantly higher in patients with CRC (39.41%) than controls (3.28%); however, 60.6% of patients with CRC still had a negative FOBT result. A positive FOBT result was associated significantly with a high risk of CRC (AOR: 16.465) (Table 2). The FOBT exhibited very strong specificity (0.9672), but its sensitivity (0.3941) was low (Table 3). Thus, we may miss roughly 60% of CRC cases if we only use the FOBT for CRC screening.

**Table 1:** Characteristics of study population with FOBT results, Cohort 1

Variable	CRC patients (N = 445)	Healthy control (N = 458)	*P*‐value
Age	63.7 ± 12.7	55.3 ± 14.7	<0.0001
Male	264 (59.33)	269 (58.73)	0.8564
BMI	23.6 ± 3.7	23.6 ± 3.2	0.9639
Stool occult blood			<0.0001
Negative	269 (60.59)	442 (96.72)	
Positive	175 (39.41)	15 (3.28)	
CRC Stage			
Stage 0	1 (0.23)	–	
Stage 1	102 (22.97)	–	
Stage 2	114 (25.68)	–	
Stage 3	166 (37.39)	–	
Stage 4	61 (13.74)	–	
Location			
Distal	324 (72.97)	–	
Proximal	120 (27.03)	–	
PlGF (pg/mL)	16.8 ± 11.4	12.0 ± 11.2	<0.0001

Note

Mean ± SD for continuous variables; N (%) for categorical variables

**Table 2:** Association between markers and the risk of CRC

	AOR	95% CI		*P*‐value
Cohort 1 (N = 903, CRC patients/healthy control = 445/458)				
Stool occult blood	16.465	9.422	28.772	<0.0001
PlGF (10 pg/mL)	1.586	1.364	1.843	<0.0001

Note

Multivariate logistic regressions were adjusted for age, sex, and BMI.

**Table 3:** CRC prediction model from Cohort 1 (N = 903, CRC patients/healthy control = 445/458)

	AUC	95% CI	*P*‐value^b^	Sensitivity	Specificity	Youden's index	PPV	NPV	FPR	FNR
Model 1	0.681	0.657‐0.705	–	0.3941	0.9672	0.3613	0.921	0.622	0.033	0.606
Model 2	0.678	0.643‐0.712	0.891	0.8131	0.4814	0.2945	0.603	0.726	0.52	0.187
Model 3	0.793	0.764‐0.821	<0.0001	0.7072	0.709	0.4162	0.703	0.714	0.291	0.293
Model 1^a^	0.756	0.724‐0.787	–	0.4247	0.9561	0.3808	0.907	0.626	0.044	0.575
Model 2^a^	0.711	0.677‐0.745	0.0047	0.8219	0.4919	0.3138	0.650	0.667	0.378	0.308
Model 3^a^	0.797	0.768‐0.825	<0.0001	0.5708	0.8614	0.4322	0.807	0.665	0.139	0.429

Note

Model 1 includes FOBT. Model 2 includes PlGF. Model 3 includes FOBT and PlGF.

Abbreviations: PPV, positive predictive value; NPV, negative predictive value; FPR, false positive rate; FNR, false negative rate

a Multivariate logistic regressions were adjusted for age, sex, and BMI.

b *P*‐value of the Model compared with Model 1

### 3.2 Serum PlGF levels was as effective as the FOBT for predicting CRC

In our previous study, we determined that the serum PlGF level was significantly higher in CRC patients than controls.^28^ Therefore, we tested the predictive power of serum PlGF level for CRC screening. Similar to our previous results, serum PlGF levels were significantly higher in CRC patients (16.8 ± 11.4 pg/mL) than controls (12.0 ± 11.2 pg/mL) (Table 1). The predictive model of serum PlGF alone was as effective as the FOBT (AUC: 0.678 vs 0.681, *P* = 0.891) (Table 3). Therefore, instead of testing FOB, measuring the serum PlGF level could be an alternative method for CRC screening; it exhibits high sensitivity (0.8131) but relatively low specificity compared with the FOBT (Table 3).

### 3.3 CRC prediction models

Next, we tested whether combining the FOBT and serum PlGF level (combined model) could improve the power of CRC screening. The combined model did prove to be a higher performing model with an AUC of 0.793 for predicting CRC, which outperformed using the FOBT (*P* < 0.0001) or serum PlGF level alone (Table 3). Both the sensitivity and specificity of the combined model were as high as 0.7072 and 0.7090, respectively. Even after adjustment for age, sex, and BMI, the combined model still had stronger predictive power for CRC than the FOBT or serum PlGF level alone (Table 3).

### 3.4 CRC prediction model stratified by CRC stages or location of CRC

To test the predictive power for early‐ (stages 0‐2) or advanced‐stage (stages 3‐4) CRC involving the proximal or distal colon (divided by the splenic flexure), we performed subgroup analysis. The total numbers of early‐ and advanced‐stage CRC patients were 217 and 227, respectively. Irrespective of whether early‐ or advanced‐stage CRC and with or without adjustment for age, sex, and BMI, our combined model still had the highest predictive power (unadjusted AUC: 0.780 for early stage and 0.805 for advanced stage; adjusted AUC: 0.797 for early stage and 0.794 for advanced stage, respectively) compared with the FOBT or serum PlGF level alone (Table 4). For both proximal‐ and distal‐site CRC and with or without adjusting for age, sex, and BMI, the predictive power of the combined model was again superior (unadjusted AUC: 0.789 for distal CRC; 0.803 for proximal CRC; adjusted AUC: 0.792 and 0.809, respectively) among these three models (Table 5).

**Table 4:** CRC prediction model stratified by CRC stage, Cohort 1

	AUC	95% CI	*P*‐value^b^	Sensitivity	Specificity	Youden's index
Early stage (N = 675, early stage CRC/healthy control = 217/458)						
Model 1	0.670	0.637‐0.704	–	0.3733	0.9672	0.3404
Model 2	0.669	0.627‐0.710	0.9514	0.8341	0.4464	0.2805
Model 3	0.780	0.743‐0.817	<0.0001	0.5668	0.8337	0.4005
Model 1^a^	0.766	0.728‐0.804	–	0.4630	0.9169	0.3798
Model 2^a^	0.718	0.679‐0.757	0.0053	0.9259	0.4388	0.3647
Model 3^a^	0.797	0.762‐0.832	0.0005	0.8009	0.6051	0.4060
Advanced stage (N = 685, advanced stage CRC/healthy control = 227/458)						
Model 1	0.692	0.658‐0.725	–	0.4159	0.9672	0.3831
Model 2	0.686	0.645‐0.726	0.8249	0.8142	0.5033	0.3174
Model 3	0.805	0.770‐0.840	<0.0001	0.5752	0.8621	0.4374
Model 1^a^	0.752	0.712‐0.792	–	0.4595	0.9284	0.3879
Model 2^a^	0.705	0.665‐0.746	0.0190	0.7568	0.5497	0.3064
Model 3^a^	0.794	0.757‐0.831	<0.0001	0.6802	0.7460	0.4261

Note

Model 1 includes FOBT. Model 2 includes PlGF. Model 3 includes FOBT and PlGF.

a Multivariate logistic regressions were adjusted for age, sex, and BMI.

b *P*‐value of the Model compared with Model 1.

**Table 5:** CRC prediction model stratified by CRC location, Cohort 1

	AUC	95% CI	*P*‐value^b^	Sensitivity	Specificity	Youden's index
Distal (N = 782, CRC patients/healthy control = 324/458)						
Model 1	0.677	0.649‐0.705	–	0.3870	0.9672	0.3542
Model 2	0.670	0.633‐0.707	0.7717	0.8142	0.4814	0.2956
Model 3	0.789	0.757‐0.821	<0.0001	0.5449	0.8621	0.4070
Model 1^a^	0.750	0.715‐0.785	–	0.4201	0.9492	0.3693
Model 2^a^	0.700	0.663‐0.737	0.0037	0.8213	0.4988	0.3202
Model 3^a^	0.792	0.760‐0.824	<0.0001	0.8025	0.6166	0.4191
Proximal (N = 578, CRC patients/healthy control = 120/458)						
Model 1	0.692	0.647‐0.737	–	0.4167	0.9672	0.3838
Model 2	0.697	0.648‐0.746	0.8890	0.6250	0.7002	0.3252
Model 3	0.803	0.758‐0.849	<0.0001	0.7417	0.7155	0.4572
Model 1^a^	0.780	0.730‐0.830	–	0.4874	0.9584	0.4458
Model 2^a^	0.730	0.681‐0.779	0.0194	0.5462	0.7829	0.3291
Model 3^a^	0.809	0.762‐0.856	0.0019	0.6134	0.8868	0.5003

Note

Model 1 includes FOBT. Model 2 includes PlGF. Model 3 includes FOBT and PlGF

a The multivariate logistic regressions were adjusted by age, sex, and BMI.

b *P*‐value of the Model compared with Model 1.

### 3.5 Additional benefit by using serum PlGF level for those FOBT negative population

Due to the high specificity and low sensitivity of FOBT, the challenges for CRC screening are focused in the FOBT negative population. Indeed, our result showed there are 269 (37.8%) CRC patients from 711 FOBT negative population. That indicates we might have missed 60.59% (269/445) of CRC cases if we had just used the FOBT to screen for CRC. However, when we added serum PlGF level to the screening protocol, we could identify an additional 50% (224/445) of CRC cases whose FOBT results were negative.

Next, we evaluated the CRC screening value of serum PlGF level in the 711 FOBT negative individuals. The adjusted AUC of predicting CRC was 0.694, and the sensitivity and specificity were 0.8195 and 0.4856, respectively (Table 6).

**Table 6:** CRC prediction value of using serum PlGF level in FOBT negative individuals (N = 711, CRC patients/healthy control = 269/442)

	AUC	95% CI	*P*‐value	Sensitivity	Specificity	Youden's index
PlGF	0.6848	0.6460‐0.7237	<0.0001	0.8327	0.4751	0.3078
PlGF^a^	0.6941	0.6550‐0.7332	<0.0001	0.8195	0.4856	0.3052

a The multivariate logistic regressions were adjusted by age, sex, and BMI

Compared with using the FOBT alone for CRC screening, the combined model increased sensitivity from 0.394 to 0.707 in unadjusted conditions and increased the sensitivity from 0.425 to 0.571 in age‐, sex‐, and BMI‐adjusted conditions (Table 3). However, the specificity decreased a little from 0.967 to 0.709 in unadjusted conditions and from 0.956 to 0.861 in age‐, sex‐, and BMI‐adjusted conditions. The positive predictive value (PPV), negative predictive value (NPV), false positive rate (FPR), and false negative rate (FNR) of the combined predictive model were 0.703, 0.714, 0.291, and 0.293 in unadjusted conditions and 0.807, 0.665, 0.139, and 0.429 in age‐, sex‐, and BMI‐adjusted conditions, respectively (Table 3). The most beneficial aspect of using the combined model might be the reduction of the false negative rate from 0.61 to 0.29 in unadjusted conditions and from 0.58 to 0.43 in age‐, sex‐, and BMI‐adjusted conditions.

### 3.6 Validation by second cohort

Because of the retrospective setting, the family histories and smoking status—two well‐known risk factors for CRC—of patients were lacking in the first cohort (cohort 1). Therefore, we performed a prospective study to include these two factors and to validate the results in our second cohort (cohort 2). Cohort 2 had 260 cases in the CRC as well as in the healthy groups. Cohorts 1 and 2 did not differ in age, sex ratio, BMI, cancer stage, or tumor location. However, similar to other reports, both smoking and a family history of CRC were significantly higher in the CRC group than the healthy group (Table S1).

Positive FOBT results and higher serum PlGF levels were both confirmed to be associated with CRC in cohort 2. The false negative rate using the FOBT was still high in CRC patients (110/260 (42.3%)) (Table S1). The AORs of the FOBT and PlGF level (10 pg/mL) were 4.72 and 1.80, respectively (Table S2). The most important finding is the reproducible high CRC screening value of serum PlGF level in FOBT negative individuals. The adjusted AUC was 0.6927, with high sensitivity (0.6727) and specificity (0.6683). Therefore, we could identify 67.3% (74/110) of CRC patients in the negative FOBT group.

The predictive power of the combined FOBT and serum PlGF level was strong even when adjusted for age, sex, BMI, family history, and smoking status (AUC: 0.772) (Table S3). Moreover, this strong predictive power was confirmed again that not influenced by CRC location or stage (Tables S4 and S5).

Finally, we divided cohort 2 into two sets: one set was the training set and the other one was the validation set. As displayed in Table 7, the AUCs of the FOBT, serum PlGF, and combined model were 0.679, 0.671, and 0.773, respectively. When we used the formula of probability derived from the training set and used the data in the validation set, we obtained a sensitivity, specificity, PPV, NPV, FPR, and FNR of 0.599, 0.734, 0.699, 0.640, 0.266, and 0.402, respectively, for the combined model (Table 7). And, in this validation set, we also noted that the sensitivity for CRC screening was higher with serum PlGF (0.659) than using the FOBT (0.583).

**Table 7:** Training and validation tests in cohort 2

	Training set (n = 260, CRC/healthy = 128/132)			Validation set (n = 260, CRC/healthy = 132/128)					
	AUC	Formula of probability	Cutoff	Sen	Spe	PPV	NPV	FPR	FNR
FOBT	0.6791	p = exp(−0.6371 + 1.5953*Stool OB)	0.7228	0.5833	0.7891	0.7404	0.6474	0.2109	0.4167
PlGF	0.6713	p = exp(−1.2150 + 0.0953*PlGF)	0.4553	0.6591	0.4844	0.5686	0.5794	0.5156	0.3409
Combined	0.7729	p = exp(−1.9485 + 1.6879*Stool OB + 0.1020*PlGF)	0.5263	0.5985	0.7344	0.6991	0.6395	0.2656	0.4015

## 4 DISCUSSION

CRC remains a major cause of mortality worldwide and survival is strongly related to the stage at diagnosis—early diagnosis is crucial to improve the likelihood of survival. Therefore, a sensitive, reliable, and cost‐effective screening tool with a high compliance rate is required for a population‐based CRC screening program. Here, we demonstrated through the use of two independent cohorts that testing the serum PlGF level was as effective as the FOBT for CRC screening. In addition, measuring serum PlGF level has high value on predicting CRC in FOBT negative populations to improve the CRC screening power, independent of the location and stage of CRC. Finally, the predictive power and sensitivity to identify CRC patients were significantly increased through the use of the combination of serum PlGF level and the FOBT, and the combination significantly decreased the false negative rate.

The FOBT is widely used for screening for CRC and can reduce CRC mortality.^35^, ^36^, ^37^ However, the test's sensitivity is low and patient compliance may be more of a problem than in checking for a blood biomarker. Blood‐based screening for CRC has been discussed and tested in recent years; however, most of the previous blood‐based markers do not exhibit high sensitivity. For example, the Septin‐9 marker was first applied to CRC screening in 2008. After a couple versions of development, several studies have evidenced that the Septin‐9 assay may not currently be applicable for adenoma detection because of its low sensitivity—the US Multi‐Society Task Force on Colorectal Cancer guidelines recommend against using it to screen for CRC.^38^ Although unsuitable for primary screening, this test could nevertheless have a limited application in patients who reject all other screening tests.

Identifying a highly sensitive blood‐based marker is a necessary to solve the problem of developing a blood‐based CRC screening strategy. Previously, we and other investigators have discovered that serum PlGF level is significantly increased in patients with CRC and thus a prognostic biomarker,^28^, ^29^, ^30^, ^31^, ^33^ but whether serum PlGF level could be used for CRC screening is unclear. In this study, we determined that the predictive power of using the serum PlGF level to screen for CRC was as effective as the FOBT and exhibited significantly greater sensitivity than the FOBT: 0.81 vs 0.39, respectively. The low specificity of serum PlGF in our cohorts might due to other conditions, such as aspirin use, cardiovascular diseases, and other tumors, which might influence the serum PlGF levels. However, as a screening test, the sensitivity is more important than specificity, Therefore, these data suggested that the serum PlGF level alone could be used as an alternative approach for CRC screening, especially for those unwilling to be screened by a stool‐based method. Unlike the stool OB‐based methods, which could be affected by the tumor location and stages; screening effect of serum PlGF was independent of the tumor location and stages.

Although the FOBT is a noninvasive and relatively low‐cost test for CRC screening, however, its sensitivity is usually low.^12^ Here we demonstrated, by using serum PlGF level, we can have a good screening effect for CRC in those FOBT negative populations. Therefore, we may use FOBT as the first line CRC screening tool since it has a good specificity. To increase the CRC screening sensitivity, we can add serum PlGF level for those FOBT negative individuals.

We also discovered that the combined model of the FOBT and a test for serum P1GF level significantly increased the predictive power of CRC screening compared with an individual test alone. The predictive power of the combined model persisted irrespective of the CRC stage or location and was confirmed after adjusting for smoking status and family history for CRC in the second independent cohort. The beneficial effect of adding a test for serum PlGF level to the FOBT was most obvious in the reduction of the false negative rate of FOB in CRC screening. We further identified 224 of 269 (83.3%) patients with CRC who had negative FOB screenings in cohort 1 by adding the serum PlGF test, and this beneficial effect was reproduced in cohort 2.

Some limitations may have affected this study. First, although we used two independent cohorts, this was still not a population‐wide screening result. We discovered that the average age was older in the patients with CRC than the healthy control group, which was consistent with our daily practice. Further large scale prospective population‐based screening is required to further confirm the results of this study. Second, we used the guaiac FOBT but not the fecal immunochemical test (FIT) for the FOB results analysis. Although we understand that FIT can exhibit higher sensitivity and specificity than the guaiac‐based chemical method (FOBT) and is recommended for CRC screening,^6^ the cost of the FIT is higher than the FOBT and it is still less widely available than the FOBT. Finally, we used the ELISA‐based method for measuring the level of serum PlGF, the cost and feasibility of which would present challenges for population‐wide screening. In the future, we expect that more rapid and cost‐effective method will become available and perhaps may even be integrated into a chip format (e.g. through the use of the surface plasmon resonance‐based biosensors).^39^

## 5 CONCLUSIONS

We demonstrated with evidence that the serum PlGF level alone can be used as an alternative CRC screening tool. By adding serum PlGF screening for FOBT negative populations, we can further improve the sensitivity of CRC screening. Combining a test for serum PlGF level and the FOBT exhibits a significant synergistic effect that can be leveraged to identify more patients with CRC and improve their outcomes.

## AUTHOR CONTRIBUTIONS

Shu‐Chen Wei: Study concept and design, data curation, writing of original draft, and investigation. Po‐Nien Tsao: Study concept and design, investigation, and review and editing. Yu‐Ting Wang: Analysis and investigation. Ben‐Ren Lin: data curation and investigation. Deng‐Chyang Wu: data curation and investigation. Wen‐Sy Tsai: data curation and investigation. Jinn‐Shiun Chen: data curation and investigation. Jau‐Min Wong: data curation, investigation, and review and editing.
