# Supplementary material for: Using serum placenta growth factor could improve the sensitivity of colorectal cancer screening in fecal occult blood negative population: A multicenter with independent cohort validation study
Source: Cancer Med. 2019 May 7;8(7):3583–91. doi: 10.1002/cam4.2216 (PMC6601572; doi:10.1002/cam4.2216)
Supplement: Supplementary file 1 [file CAM4-8-3583-s001.docx]

sTable 1. Characteristics of study population with stool occult blood results, Cohort 2

| Variable | CRC patients  (N =260) | Healthy control  (N =260) | *P*-value |
| --- | --- | --- | --- |
| Age | 61.5 ± 11.6 | 55.3 ± 12.3 | < 0.0001 |
| Male | 148 (56.92) | 157 (60.83) | 0.4229 |
| BMI | 24.2 ± 3.8 | 24.4 ± 3.6 | 0.4235 |
| Smoking status |  |  | 0.0004 |
| Never | 214 (82.31) | 228 (87.69) |  |
| Quitted | 17 (6.54) | 1 (0.38) |  |
| Smoking | 29 (11.15) | 31 (11.92) |  |
| Family history | 28 (10.77) | 11 (4.23) | 0.0046 |
| Stool occult blood |  |  | < 0.0001 |
| Positive | 150 (57.69) | 55 (21.15) |  |
| CRC Stage |  |  | NA |
| Stage 0 | 8 (3.09) | -- |  |
| Stage 1 | 47 (18.15) | -- |  |
| Stage 2 | 68 (26.25) | -- |  |
| Stage 3 | 100 (38.61) | -- |  |
| Stage 4 | 36 (13.90) | -- |  |
| Location |  |  | NA |
| Distal | 176 (68.22) | -- |  |
| Proximal | 82 (31.78) | -- |  |
| PlGF (pg/ml) | 14.1 ± 6.9 | 11.1 ± 7.1 | < 0.0001 |
|  |  |  |  |

Mean ± SD for continuous variables; N (%) for categorical variables

sTable 2. Association between markers and the risk of CRC

|  | AOR | 95% CI | | *P*-value |
| --- | --- | --- | --- | --- |
| Cohort 2 (N =520, CRC patients/healthy control = 260/260) | | | | |
| Stool occult blood | 4.72 | 3.154 | 7.063 | < 0.0001 |
| PlGF (10 pg/ml) | 1.80 | 1.354 | 2.404 | < 0.0001 |

The multivariate logistic regressions were adjusted by age, sex, BMI, family history, and smoking status (ever/no).

sTable 3. CRC prediction model from Cohort 2 (N =520, CRC patients/healthy control = 260/260)

|  | AUC | 95% CI | *P*-value^††^ | Sensitivity | Specificity | Youden’s index |
| --- | --- | --- | --- | --- | --- | --- |
| Model 1 | 0.683 | 0.644-0.722 | -- | 0.5769 | 0.7885 | 0.3654 |
| Model 2 | 0.640 | 0.592-0.687 | 0.1739 | 0.6731 | 0.5385 | 0.2115 |
| Model 3 | 0.746 | 0.704-0.789 | < 0.0001 | 0.7385 | 0.6654 | 0.4038 |
| Model 1^†^ | 0.746 | 0.704-0.788 | -- | 0.6346 | 0.7923 | 0.4269 |
| Model 2^†^ | 0.706 | 0.661-0.750 | 0.0689 | 0.7808 | 0.5423 | 0.3231 |
| Model 3^†^ | 0.772 | 0.732-0.812 | 0.0067 | 0.5577 | 0.8615 | 0.4192 |

^†^ The multivariate logistic regressions were adjusted by age, sex, BMI, family history, and smoking status (ever/no).

^††^ *P*-value of the Model compared to Model 1.

Model 1 include Stool occult blood.

Model 2 include PlGF.

Model 3 includes Stool occult blood and PlGF.

sTable 4. CRC prediction model stratified by CRC stage, Cohort 2

|  | AUC | 95% CI | *P*-value^††^ | Sensitivity | Specificity | Youden’s index |
| --- | --- | --- | --- | --- | --- | --- |
| Early stage (N = 383, early stage CRC/healthy control = 123/260) | | | | | | |
| Model 1 | 0.683 | 0.633-0.733 | -- | 0.5772 | 0.7885 | 0.3657 |
| Model 2 | 0.660 | 0.603-0.717 | 0.5521 | 0.6341 | 0.6115 | 0.2457 |
| Model 3 | 0.751 | 0.700-0.803 | < 0.0001 | 0.7724 | 0.6269 | 0.3993 |
| Model 1^†^ | 0.789 | 0.740-0.838 | -- | 0.7967 | 0.6462 | 0.4429 |
| Model 2^†^ | 0.757 | 0.707-0.807 | 0.1521 | 0.7398 | 0.6692 | 0.4091 |
| Model 3^†^ | 0.809 | 0.764-0.854 | 0.0336 | 0.6667 | 0.8346 | 0.5013 |
| Advanced stage (N = 396, advanced stage CRC/healthy control = 136/260) | | | | | | |
| Model 1 | 0.681 | 0.632-0.730 | -- | 0.5735 | 0.7885 | 0.3620 |
| Model 2 | 0.622 | 0.565-0.680 | 0.1397 | 0.7721 | 0.4154 | 0.1874 |
| Model 3 | 0.742 | 0.690-0.794 | 0.0005 | 0.7500 | 0.6692 | 0.4192 |
| Model 1^†^ | 0.717 | 0.661-0.773 | -- | 0.6176 | 0.7769 | 0.3946 |
| Model 2^†^ | 0.659 | 0.603-0.716 | 0.0624 | 0.4926 | 0.7731 | 0.2657 |
| Model 3^†^ | 0.749 | 0.697-0.800 | 0.0152 | 0.6838 | 0.7192 | 0.4031 |

^†^ The multivariate logistic regressions were adjusted by age, sex, BMI, family history, and smoking status (ever/no).

^††^ *P*-value of the Model compared to Model 1.

Model 1 include Stool occult blood.

Model 2 include PlGF.

Model 3 includes Stool occult blood and PlGF.

sTable 5. CRC prediction model stratified by CRC location, Cohort 2

|  | AUC | 95% CI | *P*-value^††^ | Sensitivity | Specificity | Youden’s index |
| --- | --- | --- | --- | --- | --- | --- |
| Distal (N = 436, CRC patients/healthy control = 176/260) | | | | | | |
| Model 1 | 0.658 | 0.614-0.703 | -- | 0.5284 | 0.7885 | 0.3169 |
| Model 2 | 0.643 | 0.591-0.695 | 0.6666 | 0.5284 | 0.6885 | 0.2169 |
| Model 3 | 0.730 | 0.683-0.778 | < 0.0001 | 0.7443 | 0.6462 | 0.3905 |
| Model 1^†^ | 0.706 | 0.655-0.756 | -- | 0.5795 | 0.7769 | 0.3565 |
| Model 2^†^ | 0.688 | 0.637-0.738 | 0.4976 | 0.6932 | 0.6654 | 0.3586 |
| Model 3^†^ | 0.744 | 0.697-0.791 | 0.0033 | 0.5909 | 0.7692 | 0.3601 |
| Proximal (N = 342, CRC patients/healthy control = 82/260) | | | | | | |
| Model 1 | 0.730 | 0.673-0.787 | -- | 0.6707 | 0.7885 | 0.4592 |
| Model 2 | 0.629 | 0.562-0.697 | 0.0260 | 0.5976 | 0.6154 | 0.2129 |
| Model 3 | 0.777 | 0.717-0.837 | 0.0115 | 0.7439 | 0.7654 | 0.5093 |
| Model 1^†^ | 0.859 | 0.815-0.903 | -- | 0.8049 | 0.8038 | 0.6087 |
| Model 2^†^ | 0.786 | 0.729-0.842 | 0.0021 | 0.8659 | 0.5731 | 0.4389 |
| Model 3^†^ | 0.861 | 0.817-0.906 | 0.6613 | 0.7683 | 0.8385 | 0.6068 |

^†^ The multivariate logistic regressions were adjusted by age, sex, BMI, family history, and smoking status (ever/no).

^††^ *P*-value of the Model compared to Model 1.

Model 1 include Stool occult blood.

Model 2 include PlGF.

Model 3 includes Stool occult blood and PlGF.
